# Hepatic Haemangopma: Enucleate or Resect?

**DOI:** 10.1155/1995/98202

**Published:** 1995

**Authors:** Dominique Franco

**Affiliations:** Hôpital Antoine Béclère 157 rue de la Porte de Trivaux Clamart Cedex 92141 France


*HPB Surgery*, 1995, Vol. 8, pp. 275–278
Reprints available directly from the publisher
Photocopying permitted by license only

© 1995 OPA (Overseas Publishers Association)
Amsterdam B.V. Published in The Netherlands
by Harwood Academic Publishers GmbH
Printed in Malaysia

**HPB INTERNATIONAL**

**EDITORIAL & ABSTRACTING SERVICE**

JOHN TERBLANCHE, EDITOR
Department of Surgery
Medical School • Observatory 7925
Cape Town • South Africa
Telephone -27-21-406-6232
Telefax -27-21-448-6461
Email: JTERBLAN@UCTGSH1.UCT.AC.ZA

### HEPATIC HAEMANGOPMA: ENUCLEATE OR RESECT?

### ABSTRACT

*Kuo, P. C., Lewis, W. D. and Jenkins, R. L. (1994) Treatment of giant hemangiomas of the liver by enucleation. Journal of the American College of Surgeons; 178: 49–53.*

**Cavernous hemangiomas are the most common benign tumors of the liver. The results of natural history studies have demonstrated that asymptomatic hemangiomas can be observed without deleterious results. The appropriate treatment for symptomatic cavernous hemangiomas remains unclear. Since 1987, ten patients with symptomatic giant cavernous hemangiomas have undergone simple enucleation at the New England Deaconess Hospital, Boston, Massachusetts. Ten patients who underwent both anatomic and nonanatomic resections for benign hepatic tumors were chosen as a control group. We analyzed patient demographics and characteristics of the hospital course. Both groups had similar periods of hospitalization (9.5 $\pm$ 1.2 versus 9.1 $\pm$ 1.8 days; p = NS), operative time (2.2 $\pm$ 0.3 versus 2.4 $\pm$ 0.2 hours; p = NS) and lesion size (7.6 $\pm$ 1.3 versus 8.4 $\pm$ 1.2 centimeters; p = NS). The enucleation group had 49 percent less intraoperative blood loss when compared with the resection group (400 $\pm$ 129 versus 742 $\pm$ 116 millimiters; $p < 0.05$). Two units of blood were transfused in the enucleation group while 6 units were transfused in the resection group. Postoperatively, two patients in the resection group required computed tomographic guided drainage of extrahepatic bile collections. There were none in the enucleation group. Because enucleation is performed in the fibrous capsule composed of compressed hepatic parenchyma, injury to major bile ducts and blood vessels may be avoided. Enucleation is a safe alternative to resection for treatment of symptomatic giant hemangiomas. *J. Am. Coll. Surg.*, 1994, 178:49–53.**

KEY WORDS: Hepatic haemangioma liver resection enucleation of hepatic haemangioma

### PAPER DISCUSSION

In this paper Kuo et al. suggest that enucleation of hemangioma is better than formal liver resection with less operative bleeding, less operative blood transfusion, and less postoperative surgical complications. The rationale of enucleation is based on the premise that there is a fibrous pseudocapsule around the hemangioma with few vessels and bile ducts crossing the capsule allowing bloodless dissection[1]. The technique used by Kuo *et al.* for enucleation includes full mobilization of the liver, clamping of the hepatic pedicle and blunt finger dissection of the hemangioma inside the pseudocapsular plane. Results of the enucleation of hemangioma in 10 patients were compared to those of formal liver resection in 10 patients with benign liver tumors, focal nodular hyperplasia or liver adenomas. Most tumors were located in the right liver. The mean diameter of hemangiomas was 7 cm and that of other tumors was 8 cm. Estimated blood loss was 46% lower in the enucleation group than in the resected group and blood transfusion was less (2 units vs 6 units). There were no intraabdominal complication after enucleation while there were 2 subphrenic abcesses after formal liver resection.

These data reflect the results previously presented both by other surgical groups[1,2] and by our own team suggesting that enucleation of liver hemangiomas is easy and safe. Is the evidence strong enough to propose enucleation as the procedure of choice in the treatment of liver hemangiomas? This should probably be tempered by a certain number of arguments. First of all a comparison between enucleation of liver hemangioma and formal liver resection for benign liver tumors might not be fair. Liver parenchyma in the vicinity of a large solid liver mass, particularly in patients with oral contraceptive induced adenomas, may be more susceptible to bleed during transection than liver parenchyma of patients with hemangiomas. Bleeding in patients with oral contraceptive induced adenomas results from marked sinusoidal congestion observed in more than 50% of these patients[3]. In the same way, it should be emphasized that formal liver resection in patients with hemangioma are amongst the easiest liver resection even when the tumor is large. Division of the artery and portal branch supplying the part of the liver to be resected markedly decreases the volume and tenseness of the tumor. As a result liver resection is usually performed as if there was not even a tumor in the liver. Blood loss during liver resection of benign liver tumors and particularly of hemangiomas should be minimal whatever the size of the tumor and the type of resection when a proper technique is used. In our own experience, only 3% of patients with liver resection for a benign tumor needed intra and/or postoperative blood transfusion[4]. The difficulty of enucleation of a tumor is quite variable depending upon its location, near the liver edge or deeply inside the liver. In the latter case, transection of liver parenchyma surrounding the tumor is a prerequisite to enucleation and the resection plane may be much larger after enucleation than after any type of anatomical liver resection. In addition, brisk bleeding may occur when the hemangioma is close to a major liver vessel. The technique of enucleation described by Kuo *et al.* includes full mobilization of the liver. As a matter of fact it has been shown that in large tumors most of intraoperative bleeding occurs during mobilization time[5]. Altogether, this suggests that there is no definite supportive evidence that enucleation of liver hemangiomas is better than formal liver resection. Small hemangiomas and hemangiomas which are located superficially may be quite easily enucleated. Large hemangiomas and those located deeply inside the liver or in the immediate vicinity of a large liver vessel (particularly a major hapatic vein) should preferably be resected by formal liver resection.

This report also rises several questions about the indications for resection of benign liver tumors and particularly of hemangiomas. Resection of hemangiomas should be considered in only a very few patients with symptomatic tumors. As in patients with gallbladder stones, the analysis of symptoms should be very cautious. In our experience, abdominal pain related to liver hemangioma is quite unfrequent. In most patients with liver hemangiomas, abdominal pain is of colonic origin. A very few patients with liver hemangioma present with an inflammatory syndrome resulting from central necrosis of the tumor[6]. Finally, the risk of rupture is quite low[7]. If resection is considered in a patient with liver hemangioma, everything should be done to avoid blood loss and blood transfusion including surgical technique, planned autotransfusion and intraoperative hemodilution[8].

### REFERENCES

1. Baer, H. U., Dennison, A. R., Mouton, W., Stain S. C., Zimmerman, A., Blumgart, L. H. (1992) Enucleation of giant hemangiomas of the liver. Technical and pathologic aspects of a neglected procedure. *Ann. Surg.*, **216**, 673–676.
2. Alper, A., Ariogul, O., Emre, A., Uras, A., Okten, A. (1988) Treatment of liver hemangiomas by enucleation. *Arch. Surg.*, **123**, 660–661.

3. Molleke, K., Stahl, E., Bretzke, G. (1979) Morphologische und Klinische Leberbefunde nach einaahme oraler Kontrazeptiva. *Z. Ges. Inn Med.*, **34**, 79–81.
4. Borgonovo, G., Bourokba, N., Vons, C., Mariette, D., Smadja, C., Franco, D. (1993) Prediction of blood transfusion during liver resection. *HPB Surgery*, **6 (suppl)** 13.
5. Bismuth, H., Castaing, D., Garden, O. J. (1989) Major hepatic resection under total vascular exclusion. *Ann. Surg.*, **210**, 13–19.
6. Pateron, D., Babany, G., Belghiti, J., et al. (1991) Giant hemangioma of the liver with pain, fever and abnormal liver tests. Report of two cases. *Dig. Dis. Sci.*, **36**, 524–527.
7. Trastek, V. F., Van Heerden, J. A., Sheedy, P. F., Adson, M. A. (1983) Cavernous hemangioma of the liver: resect or observe? *Am. J. Surg.*, **145**, 49–53.
8. Sejourne, P., Poirier, A., Meakins, J. L., Chamier, F., Smadja, C., Grange, D., Franco, D. (1989). Effect of haemodilution on transfusion requirements in liver resection. *Lancet*, **2**, 1380–1282.

Professor Dominique Franco, MD

Hôpital Antoine Béclère

157 rue de la Porte de Trivaux

92141 Clamart Cedex

France

### HPB INTERNATIONAL CORRESPONDENCE

*Editorial: Partial portacaval shunt: Narrow diameter H-graft by J. A. Myburgh.*
*Ref: HPB Surgery; 8(1): 57–59*

### COMMENT BY R. ADAM AND H. BISMUTH

Professor Myburgh's commentary makes several interesting observations about our paper. However, we would like to discuss his comment that by extrapolation of our results it can be assumed that 68% of our patients with calibrated porta caval shunts lost hepatopetal portal perfusion.

- In patients with 10 mm grafts, our data showed that hepatopetal flow was maintained at 1 year in 7 of 10 patients (70%) who underwent arteriography. Logically if these findings are extrapolated to our series of 19 patients with 10 mm grafts one would expect to find hepatopetal flow in 13 patients.
- In patients with 12 mm grafts, as Professor Myburgh states, the earlier work of Sarfeh and his colleagues[1,2] using a combination of fluoroscopy and selective angiography demonstrated that only 1 of their 12 patients receiving a 12-14 mm graft maintained pro-grade portal flow at one week after surgery. Therefore, we may assume that none of the four patients with 12 mm grafts of our series have maintained prograde portal flow.
- In patients with 8 mm grafts, as nine of the 11 patients of Sarfeh's series maintain prograde portal flow, we may assume that our two patients with 8 mm graft would have retained prograde flow.

Overall consideration of our 25 patients gives a total of 15 patients (60%) who retained prograde flow and not 32% as suggested by Professor Myburgh. Nevertheless we would agree with him that there is no absolute correlation between the occurrence of encephalopathy and maintained portal venous perfusion.

In this respect, the work of Sarfeh (2) revealed a correlation between the occurence of encephalopathy and the presence of reversed flow (35% encephalopathy in those with reversed portal flow compared with 9% in patients with prograde portal flow $p = 0.02$) but other workers have reported that factors other than straightforward preservation of pro-grade portal flow are involved in the development of post operative ancephalopathy-for example, augmentation of hepatic arterial perfusion of the liver after partial decompression[3] and maintenance of mesenteric venous hypertension limiting the absorption of nitrogenous compounds[4].

We are currently evaluating the long term results of surgery in these patients and in further patients with cirrhosis who have undergone the calibrated partial portocaval shunt. The results in our series which now stands at 43 patients tends to support the earlier work. Namely, the operative mortality rate is low (2%), the rate of variceal re-bleeding remains low (2%) and acute encephalopathy was seen in 4 patients (9%) and chronic encephalopathy in 2(5%). The actuarial survival at five years is 82%. These results augment Professor

